# Immunohistochemical staining for IMP3 in patients with duodenal papilla tumors: assessment of the potential for diagnosing endoscopic resectability and predicting prognosis

**DOI:** 10.1186/s12876-021-01811-8

**Published:** 2021-05-18

**Authors:** Hiroyuki Tanaka, Hiroki Kawashima, Eizaburo Ohno, Takuya Ishikawa, Tadashi Iida, Eri Ishikawa, Kazuhiro Furukawa, Masanao Nakamura, Takashi Honda, Yoshie Shimoyama, Ryoji Miyahara, Naoto Kawabe, Teiji Kuzuya, Senju Hashimoto, Masatoshi Ishigami, Yoshiki Hirooka, Mitsuhiro Fujishiro

**Affiliations:** 1 Department of Gastroenterology and Hepatology, Nagoya University Graduate School of Medicine, Nagoya, 4668550 Japan; 2 Department of Endoscopy, Nagoya University Hospital, 65 Tsurumai-cho, Showa-ku, Nagoya, Aichi 4668550 Japan; 3 Department of Pathology and Laboratory Medicine, Nagoya University Hospital, Nagoya, 4668550 Japan; 4 Department of Gastroenterology and Gastroenterological Oncology, Fujita Health University, Toyoake, 4701192 Japan

**Keywords:** IMP3 protein, Ampulla of Vater, Sphincter of Oddi, Pancreaticoduodenectomy, Endoscopic papillectomy, Prognosis

## Abstract

**Background:**

Endoscopic papillectomy of duodenal papillary tumors (PT) is indicated for adenomas or well-differentiated adenocarcinomas that do not involve the sphincter of Oddi. However, there is currently no reliable pre-operative method to diagnose the infiltration in the sphincter of Oddi.’ Insulin-like growth factor 2 mRNA protein 3 (IMP3) staining is reportedly associated with advanced disease stage and clinical outcomes in many carcinomas. The aim of this retrospective study was to investigate the ability of diagnosing sphincter of Oddi involvement in PT and predicting the prognoses using IMP3 immunohistochemistry.

**Methods:**

Twenty-five resected specimens from patients with PT and 24 biopsy specimens from the same patients excluding one were immunostained for IMP3. The percentage of positive cells in the tumor was evaluated and compared with the final pathological diagnosis and prognosis.

**Results:**

The final pathological diagnoses were adenoma in 5 patients and adenocarcinoma in 20 patients (no sphincter of Oddi involvement in 5 and involvement in 15). The ability to diagnose sphincter of Oddi involvement based on the percentage of IMP3-positive cells in resected specimens and tissue biopsies was the area under the curve 0.8 and 0.78, respectively, of the receiver operating characteristic curve, and the accuracies were 80.0% and 75.0% (cutoff value: 10%), respectively. Moreover, patients with an IMP3-positive cell rate of ≥ 10% had a significantly worse prognosis (log-rank test *P* = 0.01).

**Conclusion:**

IMP3 immunostaining of resected and biopsy specimens from PT patients enables the diagnosis of sphincter of Oddi involvement objectively and is also effective in predicting the prognosis.

## Introduction

Duodenal papilla adenocarcinoma is a relatively rare disease, accounting for roughly only 5% of all gastrointestinal cancers [1]. It is associated with the adenocarcinoma sequence, which is similar to that of colorectal cancer [2]. Adenoma occurs first and, in many cases, adenocarcinoma then subsequently develops in the adenoma. The rate of lymph node metastasis increases as the T-stage increases in patients with papillary adenocarcinoma. Among other studies, the rate of lymph node metastasis is 9–42% when the tumor invades the sphincter of Oddi [3, 4]. Endoscopic papillectomy is employed for early adenocarcinoma with no infiltration into the sphincter of Oddi [5] as an alternative to surgery [6]. In Japan, the Clinical Practice Guidelines for the Management of Biliary Tract Cancers (second edition and third edition) [7, 8] state that indications for localized resection by endoscopic or surgical procedures are limited to papillary adenocarcinomas without infiltration into the sphincter of Oddi. However, it is difficult to diagnose even if using imaging-based evaluation techniques such as endoscopic ultrasonography (EUS) and intraductal ultrasonography (IDUS) [9, 10]. So, pancreaticoduodenectomy (PD) is the standard treatment for papillary adenocarcinoma; however, this is a major invasive surgical procedure. Thus, preoperative confirmation of the presence of an infiltrating adenocarcinoma in the sphincter of Oddi in patients with papillary tumor (PT) is important given that such could reduce the number of patients who must undergo PD.

Insulin-like growth factor 2 messenger RNA–binding protein 3 (IMP3) is a carcinoembryonic protein [11] that plays a role in the cellular proliferation, adhesion, and infiltration of tumors in malignant neoplasms; however, its pathological role remains unclear [12]. IMP3 expression levels in esophageal adenocarcinoma are significantly associated with the depth of invasion in the context of T1a or T1b adenocarcinomas [12]. IMP3 is useful for predicting disease progression and clinical outcomes in each type of malignant tumor and in the differential diagnosis of benign and malignant tumors [13–15]. However, no study to our knowledge has yet evaluated the relationship between IMP3 and duodenal papilla adenocarcinoma.

This preliminary retrospective study evaluated whether immunochemical staining for IMP3 helps in differentiating endoscopically resectable PT (adenomas and adenocarcinomas without infiltration into the sphincter of Oddi) from papillary adenocarcinomas with an invasion into the sphincter of Oddi, which we considered the primary endpoint. This study also assessed the relationship between the immunohistochemical staining results for IMP3 and the prognosis as a secondary endpoint.

## Methods

**Fig. 1:**
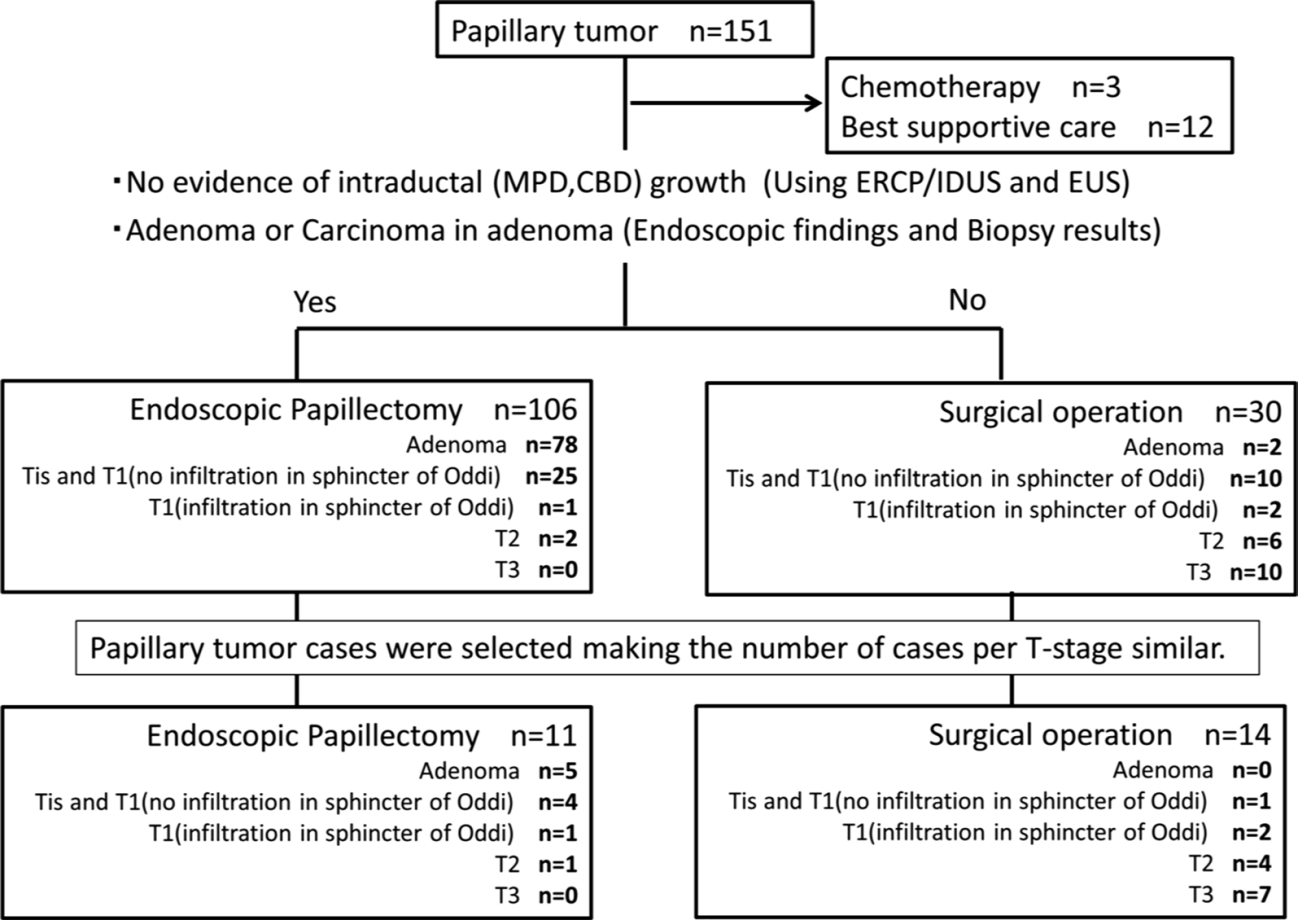
Flowchart of patient selection. In this preliminary retrospective study, 25 who could undergo sufficient pathological examination and prognostic follow-up were selected from 151 papillary tumor cases, making the number of cases per T-stage similar. *MPD* main pancreatic duct, *CBD* common bile duct, *ERCP* endoscopic retrograde cholangiopancreatography, *IDUS* intraductal ultrasonography, *EUS* endoscopic ultrasonography

Between 2012 and 2018, 151 patients with PT underwent tumor resection at Nagoya University Hospital. Of these patients, 25 who could undergo sufficient pathological examination and prognostic follow-up were selected, making the number of cases per T-stage similar [16, 17] (including 14 patients who underwent surgical resection and 11 who underwent endoscopic papillectomy; Fig. 1).

The study was approved by the hospital’s institutional review board (no. 2017-0432) and performed within the guidelines in the Helsinki Declaration for biomedical research involving human subjects (clinical trial registration number: UMIN000025631) with Grant-in-Aid for Scientific Research (grant no. 18K08614) support. Informed consent were taken from patients at the time of treatment and for full disclosure, the details of the study are published on the home page of Nagoya University Hospital and provided a means to opt out.

We first evaluated the stainability, rate of positive cells, and distribution of positive cells following immunohistochemical staining for IMP3 in specimens that were obtained by surgical or endoscopic resection. We compared these results with findings concerning the depth of invasion measured from the final pathological diagnosis and evaluated their accuracy (i.e., we determined whether IMP3 positivity indicates adenoma and adenocarcinoma without infiltration into the sphincter of Oddi with indications for endoscopic papillectomy). Next, we assessed the rate of positive cells among the 24 specimens from the same patients that were biopsied preoperatively (one patient with T3 adenocarcinoma was excluded because the biopsy had been performed at another hospital) and compared the results with diagnostic performance findings based on resected specimens. We employed the highest IMP3 positivity rate when multiple specimens were biopsied from a single patient (RadialJaw4P biopsy forceps, 2.0 mm in diameter; Boston Scientific, Natick, MA, USA).

### Immunohistochemical staining and evaluation

We conducted immunohistochemical staining for IMP3 using 4-μm-thick, formalin-fixed, paraffin-embedded tissue sections, as previously described [18, 19]. Tissue samples were fixed in 10% buffered formalin for 1 or 2 days in the biopsy specimens and from 1 to 7 days in the resected specimens. In brief, we deparaffinized the tissue sections, then incubated them in 0.3% hydrogen peroxide/methanol for 20 min at room temperature to block endogenous peroxidase activity. We performed antigen retrieval for IMP3 by heating samples in a microwave in 0.01 mol/L of citrate buffer (pH: 9.0) for 10 min at room temperature. We incubated the tissue sections with a mouse monoclonal antibody specific for IMP3 (Clone 69.1, dilution 1:100; Dako, Berlin, Germany) for 12 h at 4 °C, then further incubated them with biotinylated secondary antibodies and, finally, ABC complex (VECTASTAIN Elite ABC kit; Vector Laboratories Inc., Burlingame, CA, USA). We visualized the stained sections using diaminobenzidine and the counterstained sections with hematoxylin, respectively. We considered a case as being positive for IMP3 when the tumor cells demonstrated moderate or strong cytoplasmic IMP3-specific staining. Two researchers (Y. S. and E. I.) visually assessed the IMP3-positive cell rate independently and semiquantatively determined the percentage of IMP3-positive tumor cells, resolving any discrepancies using a multiheaded microscope. For the biopsy specimens, the entire specimen with the highest percentage of IMP-positive cells was evaluated. Of the resection specimens, those containing a large number of tumor cells were evaluated.

### Statistical analysis

We conducted all statistical analyses using BellCurve (Social Survey Research Information Co., Ltd., Tokyo, Japan) for Excel version 2.21 (Microsoft Corporation, Redmond, WA, USA). We completed a statistical analysis of group differences using the Mann–Whitney U test and receiver operating characteristic (ROC) curve for the diagnostic ability. Also, we created a Kaplan–Meier curve to evaluate the patients’ prognosis and employed a log-rank test to compare the results. A *P* value of less than 0.05 was considered to be statistically significant.

## Results

**Fig. 2:**
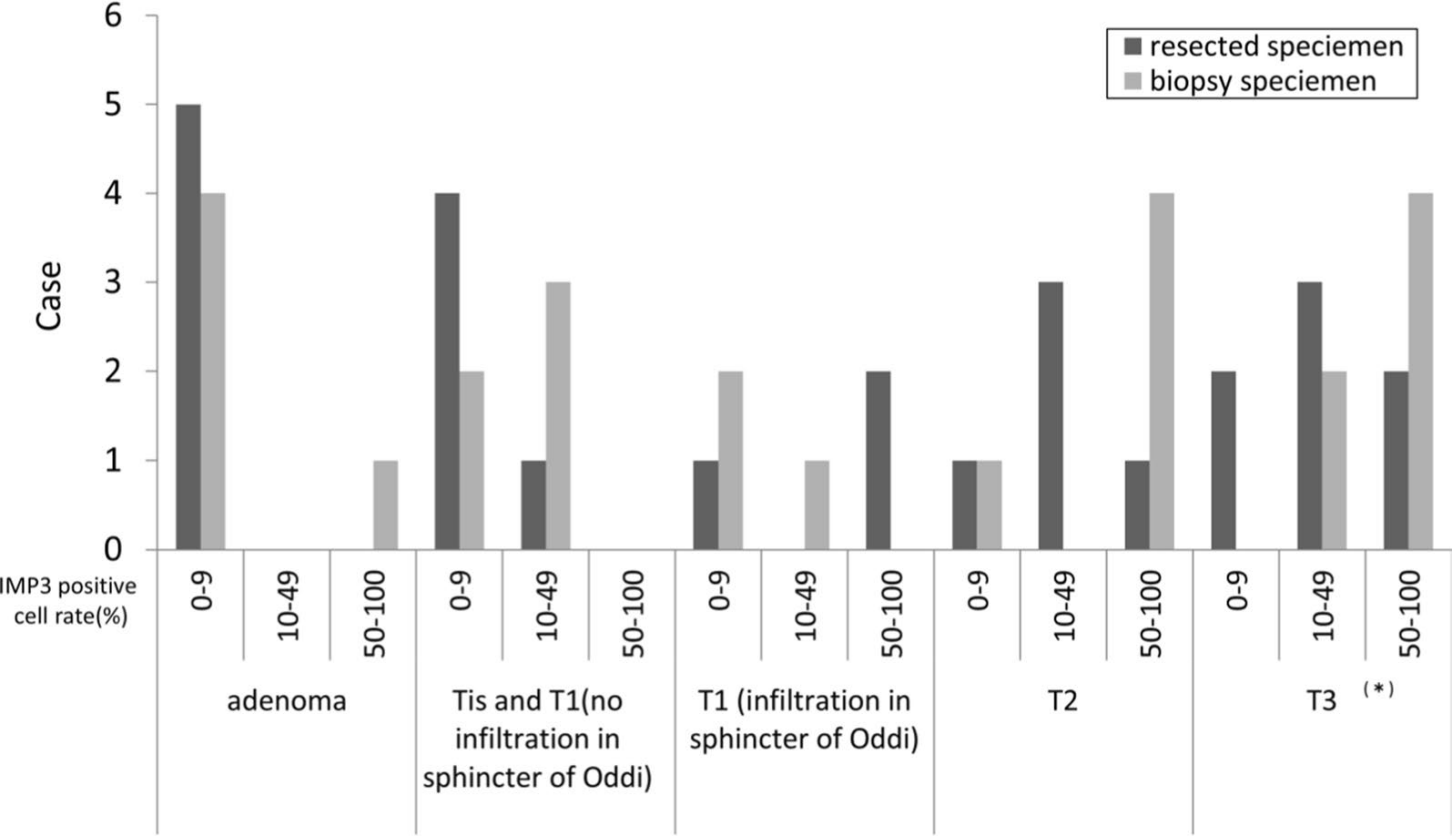
Number of cases by percentage of IMP3 positive cell rate. The rate of IMP3 positive cell between the resected specimen and the biopsy specimen is divided into 3 stages of 0–9%, 10–49%, and 50–100%, and the number of cases at each pathological diagnosis is shown. *One patient with T3 adenocarcinoma was excluded because the biopsy had been performed at another hospital. *IMP3* insulin-like growth factor 2 messenger RNA–binding protein 3

In the selected 25 patients, the pathological stages were 5, 5, 3, 5, and 7 for adenoma, Tis and T1 adenocarcinoma without infiltration into the sphincter of Oddi, T1 adenocarcinoma with infiltration into the sphincter of Oddi, T2 adenocarcinoma, and T3 adenocarcinoma, respectively (Table 1; Fig. 2). The number of T1 adenocarcinomas with infiltration into the sphincter of Oddi was limited, and all 3 patients were included. The median observation period (range) following surgical or endoscopic resection was 1347 (128–2267) days. During the observation period, five patients (two with T2 adenocarcinoma and three with T3 adenocarcinoma) died owing to their primary disease.

**Table 1:** Clinical characteristics and pathological/immunohistological findings

	Adenoma n = 5	Adenocarcinoma			
		Tis and T1	T1	T2	T3
		(no infiltration in the sphincter of Oddi) n = 5	(infiltration in the sphincter of Oddi) n = 3	n = 5	n = 7
Treatment					
Endoscopic papillectomy	5	4	1	1	0
Surgical operation	0	1	2	4	7
Tumor differentiation					
Well	–	5	2	2	0
Moderate	–	0	1	3	5
Poor	–	0	0	0	2
Lymphatic invasion	–	0	0	2	6
Venous invasion	–	0	0	2	5
Lymph node metastasis	–	0	0	2	7
Histological category					
Intestinal	5	3	3	3	1
Pancreatobiliary	0	0	0	0	4
Unclassifiable	0	2	0	2	2
IMP3-positive cells (%) in resected specimen, median (range)	5 (0–5)	5 (0–30)	50 (0–70)	20 (5–70)	20 (0–90)
IMP3-positive cell rate ≥ 10%	0	20	66.7	80	71.4
IMP3-positive cells (%) in biopsy specimens, median (range)	5 (0–80)	10 (0–30)	5 (0–60)	60 (0–100)	50 (15–100)^a^
IMP3-positive cell rate ≥ 10%	20	60	33.3	80	100^a^

*IMP3* insulin-like growth factor 2 mRNA protein 3

^a^One patient with T3 adenocarcinoma was excluded because the biopsy had been performed at another hospital

### Immunohistochemical staining for IMP3 using resected specimens

**Fig. 3:**
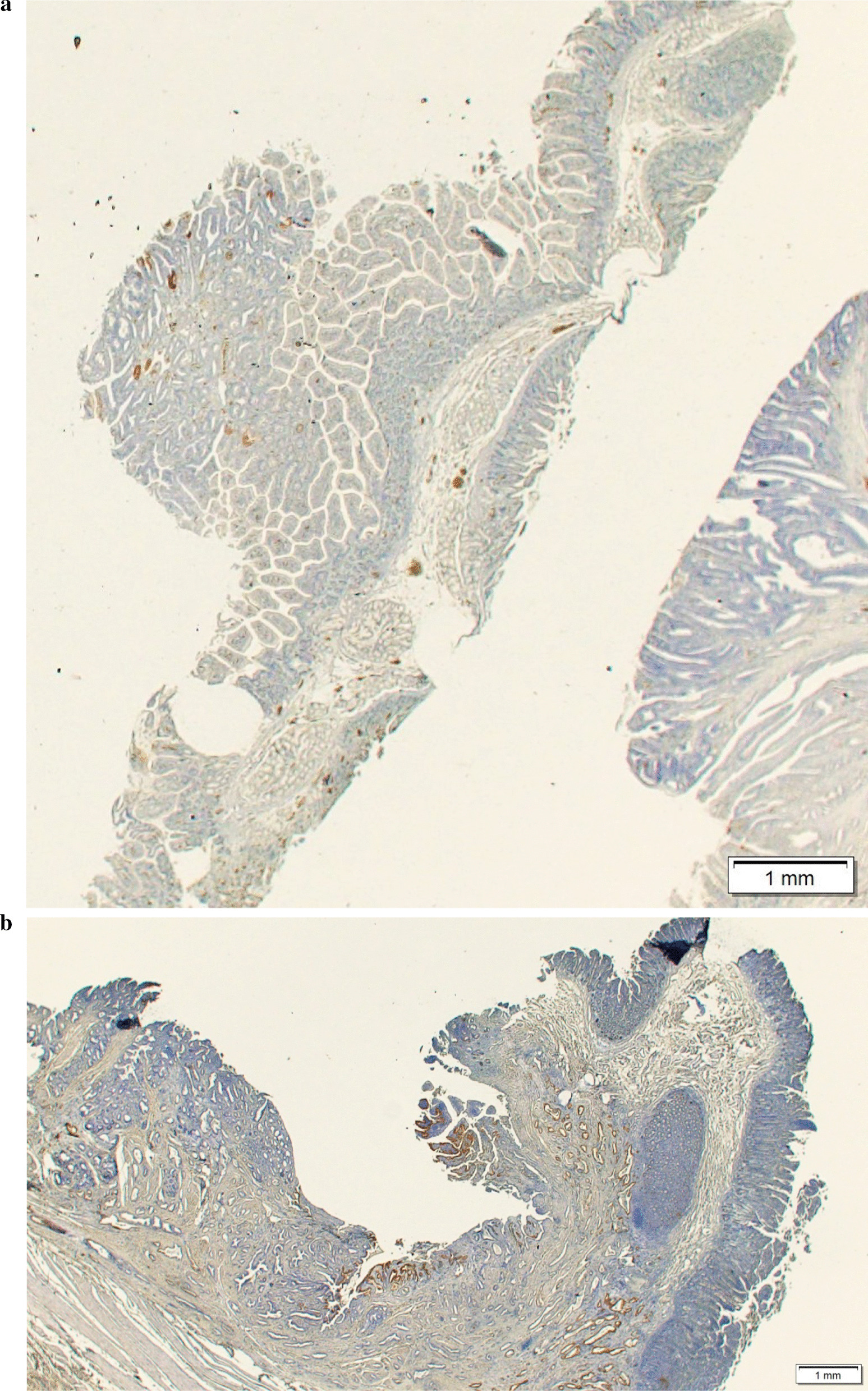

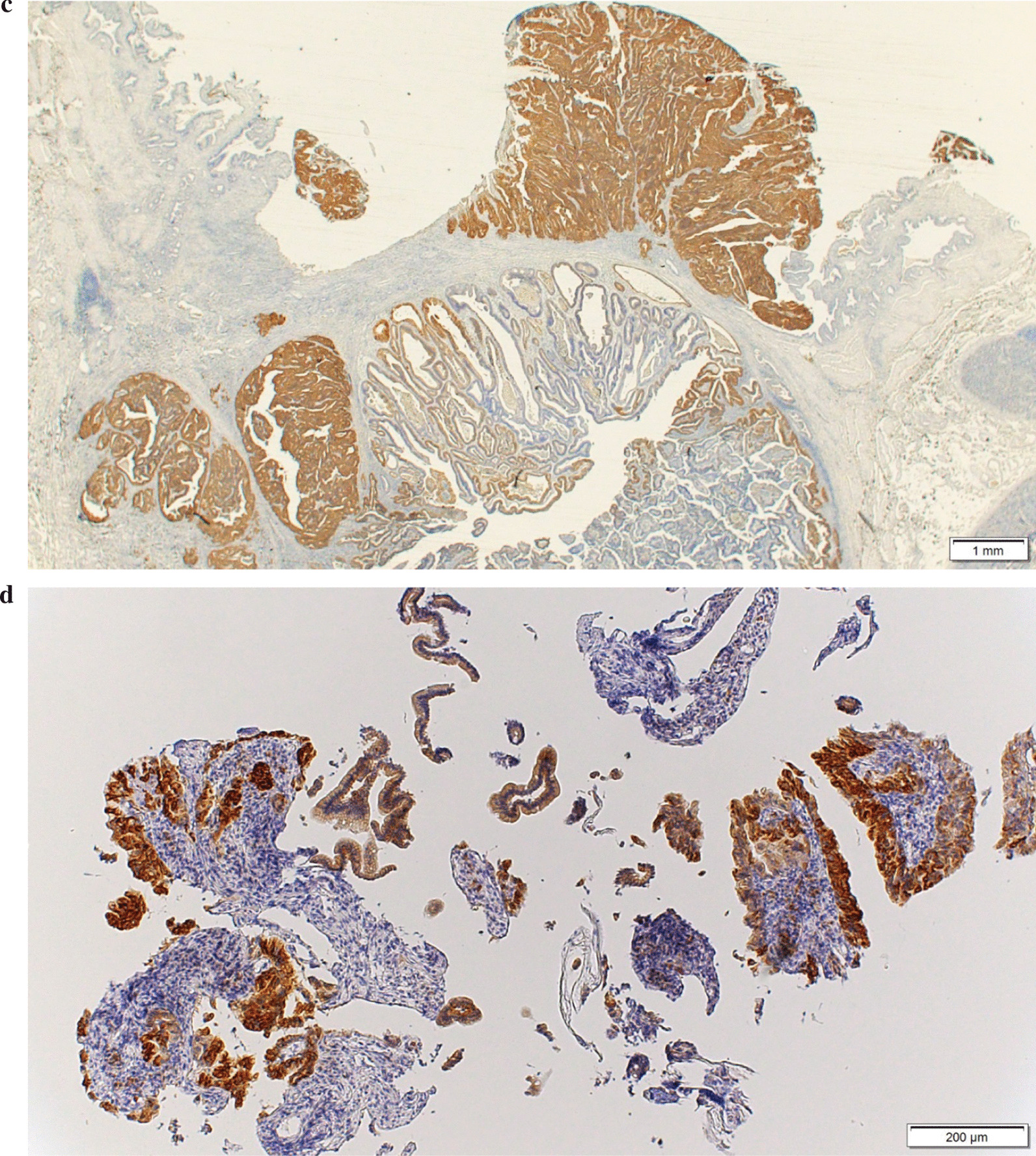
Immunohistochemical staining for IMP3 in patients with papillary adenocarcinoma. **a** A tumor endoscopically resected from a patient with T1 adenocarcinoma without infiltrating in the sphincter of Oddi: The IMP3-positive cell rate was approximately 5% in this tumor. **b** A tumor surgically resected from a patient with T2 adenocarcinoma: The IMP3 positive cell rate was approximately 20% in this tumor. **c** A tumor surgically resected from a patient with T3 adenocarcinoma: The IMP3-positive cell rate was approximately 50% in this tumor. IMP3-positive cells are expressed on the tumor surface in B and C. **d** A biopsy specimen: The IMP3-positive cell rate is approximately 80% in this sample. Surgical operation was performed, and the final pathological diagnosis was T2 adenocarcinoma. *IMP3* insulin-like growth factor 2 messenger RNA–binding protein 3

The IMP3-positive cell rate in the resected specimens was lower in the adenomas and tended to increase with T-stage in the adenocarcinomas (Table 1; Fig. 2). The distribution of IMP3-positive cells was relatively homogeneous within the tumor, regardless of whether the background tissue was adenocarcinoma or adenoma. No significant difference was found between the superficial and deeper tumor areas (Fig. 3). The median rates of IMP3-positive cells (range) were 5% (0–30%) and 20% (0–90%) in 10 patients who were eligible for endoscopic papillectomy and 15 patients with carcinoma with infiltration into the sphincter of Oddi, respectively, with a significantly high value recorded among patients with adenocarcinoma with infiltration into the sphincter of Oddi (*P* = 0.0107).

**Fig. 4:**
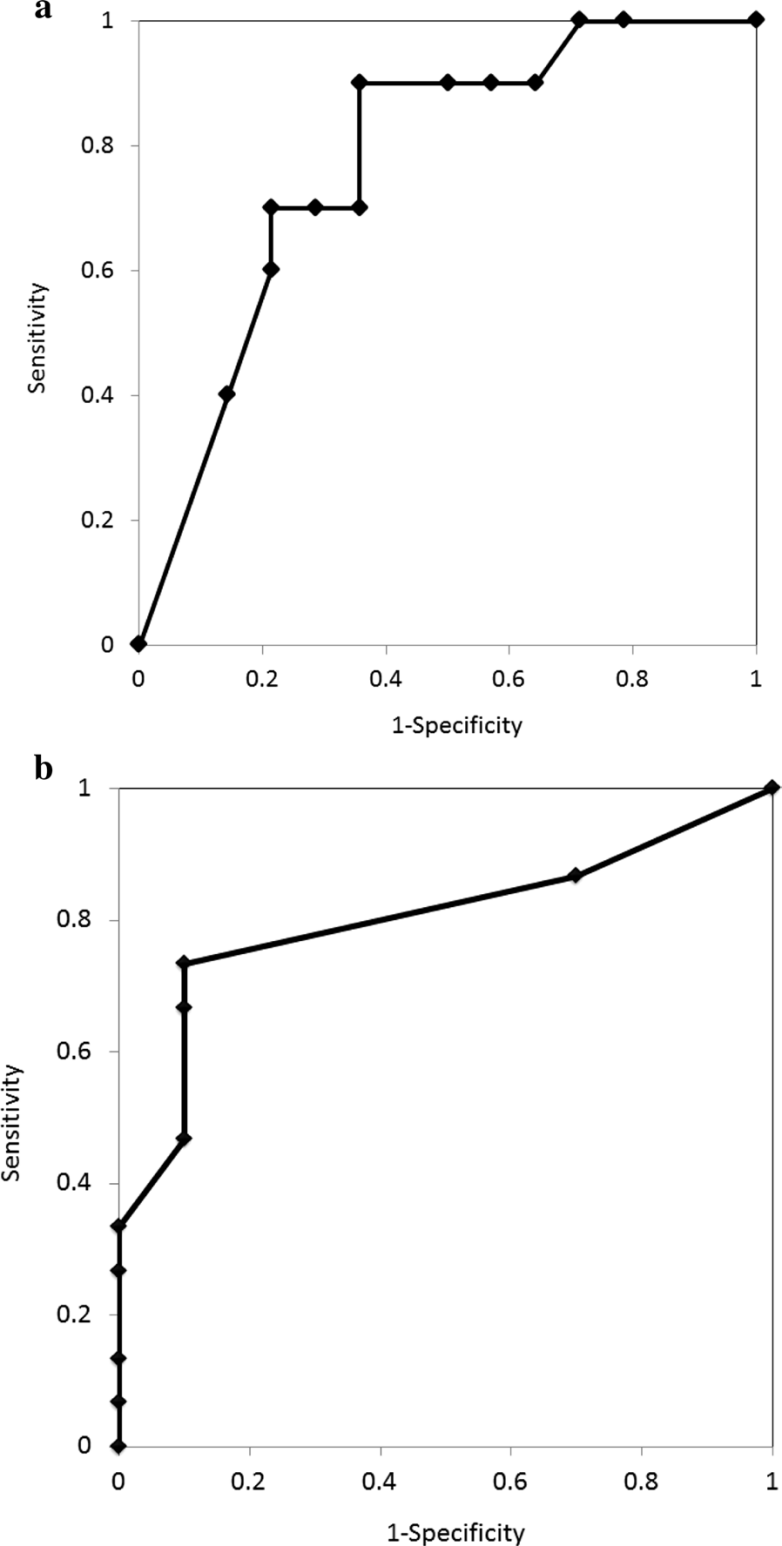
ROC curve of a resected specimen and biopsy specimen. The differential diagnostic performance based on the IMP3-positive rate was calculated as the ROC curve; specifically, patients with adenoma/adenocarcinoma (without infiltrating adenocarcinoma into the sphincter of Oddi) were indicated for localized resection and those with adenocarcinoma with invasion deeper than the sphincter of Oddi were not indicated for such a procedure, respectively. **a** The resected specimen AUC was 0.8. When the cutoff was set at 10%, the sensitivity was 73.3%, specificity was 90%, and accuracy was 80%. **b** The biopsy specimen AUC was 0.78. When the cutoff was set at 10%, the sensitivity was 70%, specificity was 78.6%, and accuracy was 75%. *IMP3* insulin-like growth factor 2 messenger RNA–binding protein 3

The diagnostic performance of endoscopic resectability based on the percentage of IMP3-positive cells was evaluated using the ROC curve. The result was favorable, with an area under the curve (AUC) of 0.8 (Fig. 4a). When the cutoff was set at 10%, its sensitivity, specificity, and accuracy values were 73.3%, 80%, and 80%, respectively.

### Immunohistochemical staining for IMP3 using preoperative biopsy specimens

The IMP3-positive cell rate in the biopsy specimens was also lower in the adenomas and tended to increase with T-stage in the adenocarcinomas (Table 1; Fig. 2).

The median IMP3-positive cell rates (range) were 5% (0–80%) and 55% (0–100%) for 10 patients with indications for endoscopic papillectomy and 14 patients with adenocarcinoma with infiltration into the sphincter of Oddi, respectively, with a significantly high value observed among patients having adenocarcinoma with infiltration into the sphincter of Oddi (*P* = 0.0228). The diagnostic performance of endoscopic resectability presented an AUC of 0.78 on the ROC curve (Fig. 4b), and when the cutoff was set at 10% (i.e., the rate of an IMP3-positive cell in a resected specimen), the sensitivity, specificity, and accuracy values were 70%, 78.6%, and 75%, respectively.

### Relationship between IMP3 immunohistochemical staining and histological type

Resected specimens were also immunostained with MUC1 and CK20 for histological typing [20]. Fifteen of our patients had the intestinal type; two, the pancreatobiliary type; and eight, the indeterminate type. The median IMP3-positive cell rates (range) of each type showed no consistent pattern at 5% (0–90%), 25% (20–30%), and 12.5% (0–80%), respectively.

### Relationship between the IMP3-positive cell rate and prognosis

**Fig. 5:**
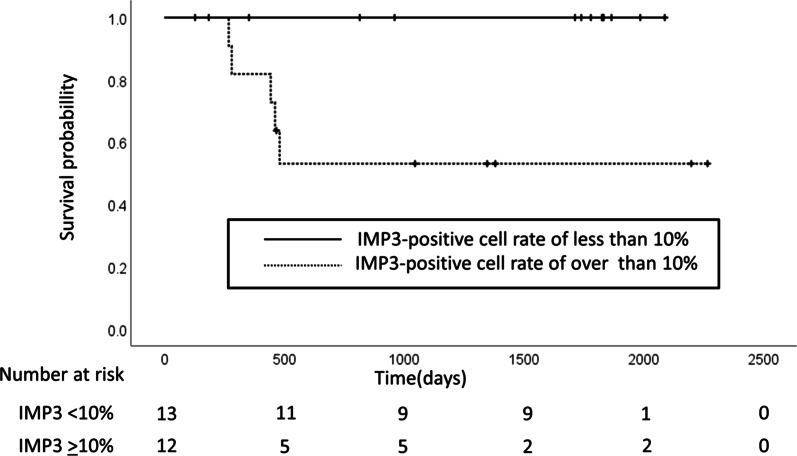
Kaplan–Meier curve. No deaths were observed among the 13 patients with an IMP3-positive cell rate of less than 10% in the resected specimens. However, five of the 12 patients with an IMP3-positive cell rate of 10% or greater in the resected specimens died. Thus, a significant association between poor prognosis and an IMP3-positive cell rate of 10% or greater was confirmed (*P* = 0.01, log-rank test). *IMP3* insulin-like growth factor 2 messenger RNA–binding protein 3

Thirteen patients had an IMP3-positive cell rate of less than 10% in their resected specimen and none of these individuals died. However, among the 12 patients with an IMP3-positive cell rate of 10% or greater in their resected specimen, five died, indicating that such a high value was significantly associated with a poor prognosis (log-rank test, *P* = 0.01) (Fig. 5).

## Discussion

A meta-analysis [21] showed that the sensitivity and specificity values of EUS for diagnosing T1 papillary adenocarcinoma were 77% and 78%, respectively. Meanwhile, the diagnostic performance of IDUS for T1 papillary adenocarcinoma is high (80–100%) [9, 22, 23]. However, EUS-based and IDUS-based imaging cannot currently detect cases of T1 adenocarcinoma infiltrating into the sphincter of Oddi; therefore, it is theoretically impossible to determine whether patients with T1 adenocarcinoma are candidates for endoscopic papillectomy using these imaging techniques. Immunohistochemical staining for IMP3 has been reported to correlate with tumor invasion and prognosis in some neoplasms. Owing to anatomical complexity, tumor invasion and prognosis are predicted to be difficult to evaluate with a single marker. Prognostic correlations with small intestinal (mostly duodenal) [11], colorectal [13], and pancreatobiliary cancers [18, 24, 25] have been reported. On the other hand, no studies using IMP3 staining for PT have been reported. In this study, IMP3 staining was expected to be an effective marker for determining the correlation with tumor invasion and malignancy in PT.

The present study determined that the presence or absence of invasive papillary adenocarcinoma in the sphincter of Oddi, which was not objectively detected through previous techniques, could be predicted with an accuracy of 75% by conducting immunochemical staining for IMP3 among biopsy specimens. Specifically, the percentage of IMP3-positive cells was low in resected specimens of adenocarcinoma without infiltration into the sphincter of Oddi, whereas this percentage was notably increased in adenocarcinoma specimens accompanied by infiltration into the sphincter of Oddi. Notably, this tendency was observed in biopsy specimens taken from the tumor’s surface and we consider it to be a very useful clinical finding. A relationship was found between the IMP3-positive cell rates in the resected and biopsy specimens for each case. However, because the specimen with the highest IMP3-positive cell rate among 1–3 biopsied tissues was adopted, the biopsy specimen tended to show a higher IMP3-positive cell rate. For clinical application, modifying the diagnosis by collecting more than three biopsy tissues and using the median IMP3-positive cell rate of all biopsy specimens may be necessary. Although only three patients experienced infiltrating T1 adenocarcinoma into the sphincter of Oddi, Two of the three patients had a high rate of IMP3 cell positivity (0%, 50% and 70%). The mechanism underlying such a high value is unclear; however, the presence of papillary adenocarcinoma invading into the sphincter of Oddi could be associated with a sudden increase in the IMP3-positive cell rate. In addition to the depth of invasion, it is speculated that tumor differentiation may be a factor affecting the rate of IMP3-positive cells. The median positive cell rates (range) were 5% (0–70%) for adenoma or well-differentiated adenocarcinoma and 30% (0–90%) for poorly or moderately differentiated adenocarcinoma, demonstrating a significant difference (*P* = 0.013).

We investigated the relationship between the presence or absence of invasion into the lymphatic and veins and the IMP3-positive cell rate among patients with adenocarcinoma. The rate tended to be higher in presence of invasion; however, there was no significant difference. The IMP3-positive cell rates in patients with T3 adenocarcinoma who died during the study were high (20%, 80%, and 90%). By contrast, these rates were relatively low among the surviving T3 adenocarcinoma patients (0%, 5%, 20%, and 30%). In addition to IMP3-positive cell rate, poorly differentiated adenocarcinoma (log-rank test *P* = 0.02) and lymphatic invasion (log-rank test *P* = 0.038) were significantly related to prognosis in the univariate analysis. However, no significant factors were observed in the multivariate analysis in this study. The IMP3-positive cell rate has been associated with a poor prognosis in other cancer types; thus, the presence of a high IMP3-positive cell rate could be a factor contributing to poor prognosis in patients with papillary adenocarcinoma. IMP3 immunostaining evaluation of specimens after endoscopic papillectomy or surgery may be useful in determining the need for adjuvant chemotherapy and follow-up duration.

The results of this study support that the treatment strategy for papillary adenocarcinoma should include endoscopic papillectomy when the IMP3-positive cell rate is less than 10% even if the patient is diagnosed with adenocarcinoma based on a pathological diagnosis using a biopsy specimen. However, this study has several limitations. First, it was retrospective and included patients from a single center. Second, the sample size was small owing to the preliminary analysis required to confirm the diagnostic performance of immunohistochemical staining for IMP3. In particular, the number of patients having T1 adenocarcinoma with invasive adenocarcinoma in the sphincter of Oddi was limited. A further prospective study involving a larger number of patients is necessary to confirm the results of this study.

In conclusion, a high IMP3-positive cell rate was found to be a predictor contributing to the poor prognosis of patients with papillary carcinoma. This study indicates that an objective diagnosis can be offered for patients with a PT to determine whether they are candidates for endoscopic papillectomy based on the IMP3-positive cell rate using a biopsy specimen.

## Data Availability

The data of this study are available from the corresponding author upon reasonable request.
